# Defects in skeletal muscle subsarcolemmal mitochondria in a non-obese model of type 2 diabetes mellitus

**DOI:** 10.1371/journal.pone.0183978

**Published:** 2017-08-29

**Authors:** Nicola Lai, China Kummitha, Charles Hoppel

**Affiliations:** 1 Department of Electrical and Computer Engineering, Old Dominion University, Norfolk, Virginia, United States of America; 2 Biomedical Engineering Institute, Old Dominion University, Norfolk, Virginia, United States of America; 3 Department of Biomedical Engineering, School of Engineering, Case Western Reserve University, Cleveland, Ohio, United States of America; 4 Department of Pharmacology, School of Medicine, Case Western Reserve University, Cleveland, Ohio, United States of America; 5 Center for Mitochondrial Disease, School of Medicine, Case Western Reserve University, Cleveland, Ohio, United States of America; 6 Department of Medicine, School of Medicine, Case Western Reserve University, Cleveland, Ohio, United States of America; Universidad Pablo de Olavide, SPAIN

## Abstract

Skeletal muscle resistance to insulin is related to accumulation of lipid-derived products, but it is not clear whether this accumulation is caused by skeletal muscle mitochondrial dysfunction. Diabetes and obesity are reported to have a selective effect on the function of subsarcolemmal and interfibrillar mitochondria in insulin-resistant skeletal muscle. The current study investigated the role of the subpopulations of mitochondria in the pathogenesis of insulin resistance in the absence of obesity. A non-obese spontaneous rat model of type 2 diabetes mellitus, (Goto-Kakizaki), was used to evaluate function and biochemical properties in both populations of skeletal muscle mitochondria. In subsarcolemmal mitochondria, minor defects are observed whereas in interfibrillar mitochondria function is preserved. Subsarcolemmal mitochondria defects characterized by a mild decline of oxidative phosphorylation efficiency are related to ATP synthase and structural alterations of inner mitochondria membrane but are considered unimportant because of the absence of defects upstream as shown with polarographic and spectrophometric assays. Fatty acid transport and oxidation is preserved in both population of mitochondria, whereas palmitoyl-CoA increased 25% in interfibrillar mitochondria of diabetic rats. Contrary to popular belief, these data provide compelling evidence that mitochondrial function is unaffected in insulin-resistant skeletal muscle from T2DM non-obese rats.

## Introduction

Muscle metabolic function, vital in maintaining health and quality of life, declines in type 2 diabetes mellitus (T2DM), accompanied by mitochondrial dysfunction and insulin resistance (IR). Skeletal muscle IR plays a major role in the pathogenesis of T2DM, because, during the postprandial state, 65–80% of whole body glucose uptake takes place in skeletal muscle stimulated by insulin [1]. Resistance of skeletal muscle to insulin is related to an accumulation of lipids, which appears to impair the insulin signaling pathway [2] that triggers glucose uptake in the muscle. The cause of the accumulated lipid-derived products (or lipid derivatives) is less clear and there still is a debate as to whether skeletal muscle mitochondria have a primary or secondary role in this accumulation [3,4].

Both human and animal model studies provide conflicting results concerning the primary or secondary role of skeletal muscle mitochondria in determining IR [5,6]. It is not clear whether lipid accumulation is caused by mitochondrial dysfunction rather than by enhanced fatty acid transport and/or overload [4, 7, 8]. T2DM is typically associated with obesity; skeletal muscle mitochondrial function, electron transport chain (ETC) activity, fatty acid transport, and β-oxidation function have been investigated under IR and obese conditions [6, 9, 10]. These investigations have been extended to lean and obese conditions without IR to determine the causal relationship between IR, fat overload, and mitochondria energy metabolism during the progression of the disease [11, 12, 13].

In obese T2DM patients, bioenergetics studies on permeabilized skeletal muscle fibers [5, 14] showed lower respiration rate than that measured in the control group. But respiration rate normalized to citrate synthase as a biomarker of mitochondria content was similar in diabetic and control subjects. In contrast to the view of normal mitochondria function, another study [6] reported reduced complex I activity in skeletal muscle mitochondria isolated from frozen biopsies from obese T2DM patients. In obese nondiabetic subjects, palmitate oxidation was reduced in muscle strips [9] while not altered in isolated mitochondria [12]. In the former study, citrate synthase was not measured while in the second study citrate synthase was reduced in muscle of obese women [12]. This difference could be attributed to the reduced mitochondrial content within the muscle strip as reported for the permeabilized fibers studies on diabetic patients [5, 14]. Another skeletal muscle mitochondria study also reported similar palmitoylcarnitine oxidation in obese nondiabetic and T2DM patients [10]. Consistent with the notion of a decreased mitochondrial content in muscle of obese patients, a study on leg substrate oxidation using arteriovenous measurements, observed a reduced capacity to oxidize fatty acid in obese subjects [6]. In the latter study, muscle carnitine palmitoyltransferase (CPT1) activity was reduced in obese individuals. CPT1 is a mitochondrial enzyme that catalyzes the conversion of fatty acyl-CoA to acylcarnitine, which can be transported into mitochondria for β-oxidation. In lean insulin-resistant individuals, alteration of skeletal muscle metabolism involved a 30% decrease in muscle substrate oxidation in the presence of elevated intramyocyte lipid and plasma fatty acid concentrations [11].

A high fat diet animal study using skeletal muscle and isolated mitochondria suggested that mitochondria fatty acid oxidation was increased in the insulin-resistant rodent [15]. Moreover, in a similar study, an increase of incomplete fatty acid oxidation was found to contribute to skeletal muscle insulin resistance [16]. Also, a high fat diet was reported to increase mitochondria proton leak and capacity to oxidize fatty acid although this capacity was not increased in skeletal muscle [17]. The accumulation of fatty acid oxidation intermediates appears to cause mitochondrial uncoupling with ATP production inefficiency.

Skeletal muscle mitochondria exist in at least two populations, subsarcolemmal (SSM) and interfibrillar (IFM); diabetes and obesity have been reported to selectively affect SSM rather than IFM in determining alterations of bioenergetic function. In a human study, the relative succinate oxidase activity was selectively reduced in SSM rather than in IFM of skeletal muscle of obese and T2DM populations [18]. In the same study, electron transport chain (ETC) activity of SSM was reduced in diabetic patients compared to active lean adults. In obese Zucker rat skeletal muscle, fatty acid transport, esterification, and oxidation were enhanced in SSM, but unaltered in IFM [19]. In contrast, another study reported that the respiratory capacity of SSM was reduced and that of IFM was preserved in skeletal muscle of rats fed a high fat diet [13]. Thus, the cause for the discrepancies between human and animal model studies is not resolved. The role of the subpopulations of mitochondria in the pathogenesis of IR in the absence of obesity has not been dealt with. Therefore, in this study, a non-obese and spontaneous rat model of T2DM, Goto-Kakizaki (GK), was used to evaluate bioenergetic function in both populations measuring integrated mitochondria function, as well as β-oxidation and ETC activity. Our proposal was that in the absence of obesity in insulin-resistant rats, bioenergetic function and biochemical properties of the ETC as well as fat oxidation are not altered in skeletal muscle mitochondria, both SSM and IFM.

## Methods

### Materials

Reagents: Dispase, trypsin and collagenase type 2 were purchased from Worthington Biochemical Corporation (Lakewood, NJ. Unless otherwise specified, all other reagents were obtained from Sigma-Aldrich (St Louis, MO, USA).

### Buffers

The buffers, Chappell–Perry (CP) (100 mM KCl, 50 mM MOPS, 5 mM MgSO_4_, 1mM ATP) [20], CP2 (Buffer CP plus 0.2% defatted BSA and 1 mM EGTA), KME (100 mM KCl, 50 mM MOPS and 0.5 mM EGTA, pH 7.4) were prepared for tissue storage, mitochondrial isolation, and mitochondria storage [21]. The respiration buffer (80 mM KCl, 50 mM MOPS, 1 mM EGTA, 5 mM KH_2_PO_4_, and 1 mg/mL defatted BSA, pH 7.0) was used for mitochondrial oxygen uptake measurements [22].

### Animal model

The experimental protocols conformed to the Guide for the Care and Use of Laboratory Animals published by the National Research Council [23] and were approved by the Case Western Reserve University Institutional Animal Care and Use Committee. A non-obese model of type 2 diabetes mellitus (T2DM), Goto-Kakizaki (GK) rats, and Wistar colony rats as a control group were obtained from Charles River. Twelve male GK and 12 male Wistar (W) rats were housed in pairs in the Animal Resource Center facilities of Case Western Reserve University under a 12:12-h light-dark cycle and were fed a standard diet chow (Prolab Isopro RMH 3000, LabDiet, St. Louis, MO). The GK and W rats were euthanized by decapitation using a guillotine at 18 wk (n = 6) and 28 wk (n = 6) of age.

### Isolation of mitochondria

Skeletal muscle SSM and IFM were isolated from quadriceps muscle (2–4 g wet tissue) using a previously developed [24, 25] protocol with minor modifications. After fat and connective tissue were removed, the skeletal muscle was blotted dry and weighed. To separate fibers, the tissue was minced and resuspended in cold (4°C) buffer (CP) (5 ml/ g wet tissue), and incubated with 0.18 mg dispase per gram wet tissue in CP buffer with stirring on ice for 10 min. Subsequently, 10 ml of CP2 buffer/g wet tissue was added to the sample and centrifuged at 7650g for 10 min. The pellet was resuspended in CP2 buffer (5 ml/ g wet tissue) and homogenized at 400 rpm (Fisher Maxima Overhead Stirrer) with a loose pestle (Potter-Elvehjem) and centrifuged for 10 min at 580 g. The supernatant was decanted from the myofibrillar pellet and centrifuged at 7000 g for 20 min to collect the SSM fraction. The myofibrillar pellet was resuspended in CP buffer (10 mL/ g wet tissue), treated with collagenase 2 (30 mg/g wet tissue) and trypsin (5 mg/g wet tissue) for 10 minutes on ice with stirring, and homogenized at 1600 rpm with a tight-fitting pestle (Potter-Elvehjem). An equal volume of CP2 buffer was added to the homogenate and centrifuged for 10 min at 12,000 g. The pellet was resuspended in CP2 buffer (5 ml/g wet tissue) and centrifuged for 10 min at 350 g. To isolate the IFM fraction, the supernatant was collected by filtering through two layers of gauze and centrifuged at 7000 g for 20 min. Both SSM and IFM fractions were washed first with 5 ml/g wet tissue of CP2 buffer and second with 2.5 ml/g wet tissue of KME buffer (BSA free). The KME buffer was used to resuspend the final pellets to a concentration of ~30–40 mg mitochondrial protein/ml. Ice-cold conditions were maintained throughout the SSM and IFM isolation procedure. The Lowry method was used to determine mitochondrial protein concentration [24].

### Oxidative phosphorylation

Oxygen consumption of SSM and IFM was measured with a Clark-type electrode (YSI model 53) in a final volume of 0.5 mL of respiration buffer [22, 26] at 30°C in the presence of the following substrates and inhibitors: glutamate (G, 20 mM), pyruvate (P, 10 mM) plus malate (M, 5 mM), glutamate (G, 20 mM) plus malate (M, 5 mM), succinate (S_R_, 20 mM) plus rotenone (7.5 μM), duroquinol (DHQ_R_, 1 mM) plus rotenone (7.5 μM), N,N,N′,N′-tetramethyl-p-phenylenediamine (TMPD, 1 mM) plus ascorbate (A, 10 mM) plus rotenone (TMPD+A)_R_, 7.5 μM, palmitoylcarnitine (PCN, 40μM) plus malate (M, 5 mM), palmitoyl-CoA (P-CoA, 20μM) plus malate (M, 5 mM) and plus carnitine (C, 5mM) assays. The concentrations of substrates, inhibitor, uncoupler, and ADP refer to the final concentrations in the chamber. The assays with complex II, III, and IV substrates were performed with rotenone to inhibit complex I. ADP at a concentration of 0.1 mM was used to deplete endogenous substrates before adding the substrates G, P+M, G+M, PCN+M or P-CoA+M+C, and 0.2 mM ADP (ADP-stimulated) to measure State 3 respiration rate. For only S, DHQ, and TMPD+A assays ADP was added to 0.1 mM (ADP-stimulated). After ADP depletion, State 4 respiration was measured (ADP-limited). The mitochondria State 3 and 4 respiration rates were measured twice for each assay. The respiration rate was then measured in the presence of high ADP concentration, 2 mM ADP. Finally, 0.2 mM of the uncoupler dinitrophenol (DNP) was added to the chamber [25] to measure the oxidative capacity of mitochondria. The respiration rate is reported in nAO min^-1^ mg^-1^ while the conversion factor to express the respiration rate in pmolO_2_ s^-1^ mg^-1^ is 8.333.

Respiratory control ratios (RCR, State 3 divided by State 4) are calculated to quantify the control of oxygen consumption by phosphorylation (‘coupling’). The concentration of ADP was determined by an enzymatic method [21] and used to calculate the ADP/O ratio (ADP mole added for mole of oxygen atom consumed), an index of the efficiency of oxidative phosphorylation [27].

### Preparation of samples and enzymatic assays

Mitochondrial enzyme activities were measured as described previously [22, 26, 28, 29]. Briefly, fresh SSM and IFM (1 mg of mitochondrial protein) samples were treated with cholate (10 mg cholate /mg of protein) [30] in 25 mM KPi/2mM EDTA buffer to a final volume of 1 mL supplemented with mammalian protease inhibitor cocktail (10 μL/ mg). The samples were diluted 1:10 for the enzymatic assays. For cytochrome c oxidase (CIV) activity, 0.1 mg of fresh intact mitochondria were suspended in 25 mM KPi/2mM EDTA buffer + protease inhibitor and assayed with dodecyl D-maltoside in the cuvette with and without KCN; the first-order rate constant was determined and the cyanide sensitive activity recorded. ETC enzyme activities were measured by specific donor–acceptor oxidoreductase activities in 0.1 M phosphate buffer using a spectrophotometer: CI, complex I–rotenone-sensitive; CIII, complex III—antimycin A-sensitive; NCR, rotenone-sensitive NADH-cytochrome c reductase; SCR, antimycin A-sensitive succinate-cytochrome c reductase; CS, citrate synthase; NFR, NADH-ferricyanide reductase; SDH, succinate dehydrogenase; Aconitase; CII, TTFA sensitive complex II; CII+Q, TTFA sensitive complex II with exogenous coenzyme Q analogue. The donors and acceptors span specific regions of the ETC [26, 28, 29]. The ETC activity components were determined using biochemical kinetics principles. Cytochrome *c* oxidase activity also was determined by a polarographic assay using the protocol for kidney [31] and heart [32] mitochondria.

Skeletal muscle tissue samples were used to determine citrate synthase (CS) and succinate dehydrogenase (SDH) activity and were treated with cholate (10 mg cholate/10 mg) in 25 mM KPi/2mM EDTA buffer to a final concentration of 10 mg wet weight tissue/mL supplemented with mammalian protease inhibitor cocktail (10 μL/ mL). Citrate synthase activity and succinate dehydrogenase were measured using the spectrophotometer at 412 and 600 nm, respectively [26, 33]. Mitochondrial cytochrome contents (*aa*_*3*_, *b*, *c*_*1*_, *c*) were determined using the method of Williams [34].

### Statistical analysis

Results are reported as means ± standard deviation. Differences between control and diabetic rats during the time course were compared by one-way analysis of variance. A difference of P < 0.05 was considered significant.

## Results

### Animal model

The characteristics of the animal model are reported in Table 1. The body weight of diabetic (GK) rats is significantly lower than that of control (W) rats at both 18 and 28 weeks. The body weight of GK rats does not change from 18 to 28 weeks, while that of W rats significantly increases by 24%. The GK rats are hyperinsulinemic and hyperglycemic at 18 and 28 weeks (Table 1).

**Table 1:** Animal characteristics: Body weight, insulin and glucose concentration in blood.

	Unit	W	GK	W	GK
		18 wk		28 wk	
**Body weight**	[g]	474±47	350±23*	590±58	389±21^#^
**Insulin**	[ng mL^-1^]	2.8±1.4	5.2±2.1*	2.4±2.1	5.1±1.4^#^
**Glucose**	[mM]	6±1	16.5±2.3*	6.5±1.7	17.4±3^#^

(n = 6)

* (P<0.03) W-18wk vs. GK-18 wk;

^#^ (P<0.03) W-28wk vs. GK-28 wk;

### Skeletal muscle mitochondria

To quantify mitochondrial content in skeletal muscle, the enzyme activities of CS and SDH are used as mitochondrial markers. At 18 weeks, both CS and SDH activity are 15–25% lower in skeletal muscle of diabetic rats compared to controls, but no difference is observed between those two groups at 28 weeks (Table 2). This suggests a lower mitochondrial content in skeletal muscle of only diabetic rats at 18 weeks than that at 28 weeks. The specific activity of CS and SDH in the isolated mitochondrial is similar in the two groups of rats at both ages. The yield for SSM and IFM of diabetic rats is 20% lower than from control rats only at 18 weeks (Table 2). These yields in GK and W groups are consistent with the lower CS and SDH activity in GK versus W rats at 18 weeks.

**Table 2:** Specific activities of mitochondrial enzymes in skeletal muscle homogenate (U g^-1^ wet weight) and isolated subsarcolemmal (SSM) and interfibrillar (IFM) mitochondria (U g^-1^ mitochondrial protein).

		Unit	W	GK	W	GK
			18 wk		28 wk	
	**Skeletal muscle homogenate**					
**Citrate Synthase**		[U g^-1^]	40±6	33±3*	35.5±3	39.6±6
**Succinate Dehydrogenase**		[U g^-1^]	3.2±0.3	2.4±0.3*	3.8±0.5	3.9±0.6
	**Isolated mitochondria**					
**Citrate Synthase**	**SSM**	[U g^-1^]	1714±143	1658±112	1753±163	1646±303
	**IFM**	[U g^-1^]	1915±140	1929±272	2088±234	2208±65
**Succinate Dehydrogenase**	**SSM**	[U g^-1^]	224±24	220±27	232±20	206±35
	**IFM**	[U g^-1^]	242±21	254±46	275±18	288±32
**Aconitase**	**SSM**	[U g^-1^]	579±196	627±53	690±71	600±86
	**IFM**	[U g^-1^]	593±105	695±75	826±99^¥^	750±93
	**Mitochondrial Yield**					
	**SSM**	[mg g^-1^]	3.5±0.4	2.8±0.3*	3.1±0.6	2.8±0.4
	**IFM**	[mg g^-1^]	8.7±1.4	7.0±0.9*	6.9±1^¥^	6.5±1

(n = 6)

* (P<0.05) W-18wk vs. GK-18 wk;

^¥^ (P<0.05) W-18wk vs. W-28 wk;

Aconitase activity measures mitochondrial matrix oxidative stress. The aconitase specific activity is similar in both populations of mitochondria from control and diabetic group of rats. In control rats, aconitase activity is 30% higher at 28 wk than at 18 wk.

### Oxidative phosphorylation

**Fig 1:**
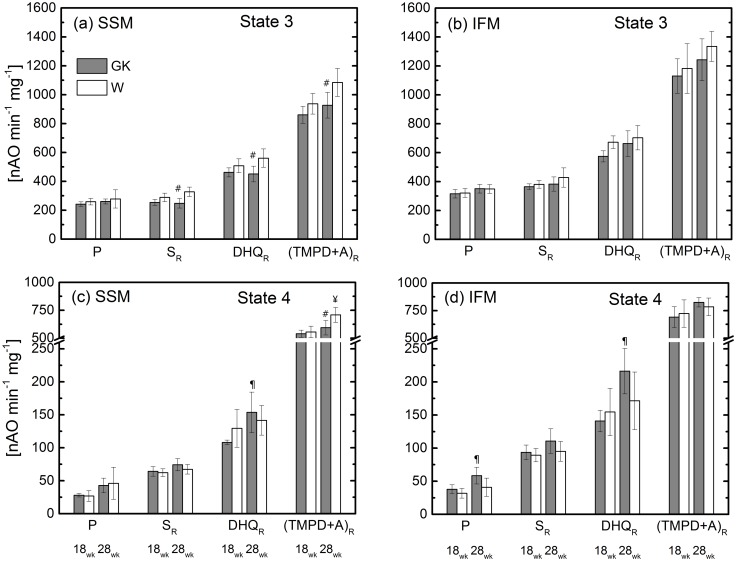
State 3 (a and b) and state 4 (c and d) respiration rates of skeletal muscle SSM and IFM at 18 and 28 weeks. (Control (W) and diabetic (GK) groups are designated with open and grey bars, respectively). Complex I substrate (malate and pyruvate, P); Complex II (succinate and rotenone, S_R_); Complex III (duroquinol and rotenone, DHQ_R_); Complex IV (TMPD, ascorbate and rotenone, (TMPD+A)_R_. ^¥^(P<0.05) W-18wk vs. W-28; ^¶^(P<0.05) GK-18wk vs. GK-28; ^#^(P<0.05) control W-28wk vs. diabetic GK-28; (n = 6), Mean ± SD.

**Fig 2:**
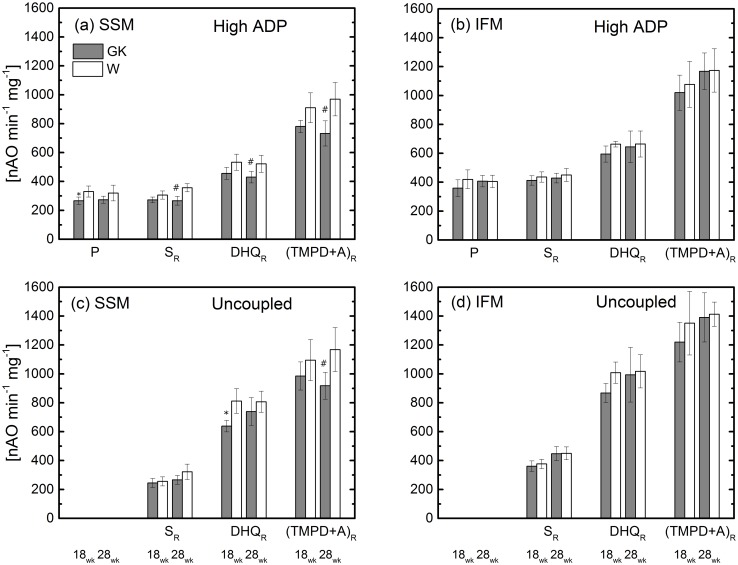
ADP saturated concentration (a and b) and uncoupled (c and d) respiration rates of skeletal muscle SSM and IFM at 18 and 28 weeks. Notation as in Fig 1. Complex I substrate (malate and pyruvate, P); Complex II (succinate and rotenone, S_R_); Complex III (duroquinol and rotenone, DHQ_R_); Complex IV (TMPD, ascorbate and rotenone, (TMPD+A)_R_. *(P<0.05) W-18wk vs. GK-18 (n = 6); ^#^(P<0.05) W-28wk vs. GK-28; (n = 6), Mean ± SD.

**Fig 3:**
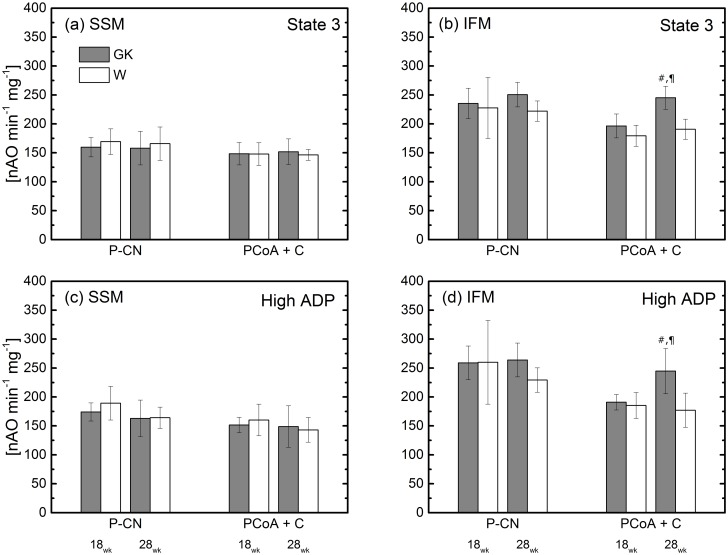
ADP unsaturated (a and b) and saturated concentration (c and d) respiratory rates of lipid substrates in skeletal muscle SSM and IFM at 18 and 28 weeks. Notation as in Fig 1. Malate and palmitoylcarnitine, PCN; malate, palmitoyl-CoA and carnitine (P-CoA+C). ^¶^(P<0.05) GK-18wk vs. GK-28; ^#^(P<0.05) W-28wk vs. GK-28; (n = 6), Mean ± SD.

The rates of oxidative phosphorylation for SSM and IFM are measured at 18 and 28 weeks of age in both GK and W rats (Table 3 and Figs 1–3). In both populations of mitochondrial, the RCR of the W and GK group of rats in both age groups is higher than 15 when glutamate is used as substrate. The RCR values indicate that both populations of mitochondrial are highly coupled in the diabetic and control rats at both ages.

**Table 3:** Oxidative phosphorylation using glutamate in skeletal muscle subsarcolemmal mitochondria (SSM) and interfibrillar mitochondria (IFM) in control (W) and diabetic (GK) rats.

	Unit	W	GK	W	GK
		18 wk		28 wk	
		**SSM**			
**State 3**	[nAO min^-1^ mg^-1^]	297±22	257±26*	305±22	279±22
**State 4**	[nAO min^-1^ mg^-1^]	14±5.5	12.7±4.4	12.6±4	17.3±6
**RCR**	[-]	26.5±16.1	21.9±5	27±10	18.0±5.6
**ADP/O**	[-]	2.96±0.2	2.90±0.21	2.88±0.17	2.70±0.14
**Maximal ADP**	[nAO min^-1^ mg^-1^]	409±31	380±48	384±56	361±31
**DNP**	[nAO min^-1^ mg^-1^]	446±35^§^	421±63^§^	439±66^§^	407±34^§^
		**IFM**			
**State 3**	[nAO min^-1^mg^-1^]	365±27	339±39	356±37	363±36
**State 4**	[nAO min^-1^mg^-1^]	18.6±4.7	22.4±6.3	15.4±10	18.9±4.6
**RCR**	[-]	21.5±7.5	16.3±5.4	30.7±15.7	20.7±5.7
**ADP/O**	[-]	2.99±0.11	3.04±0.26	2.84±0.25	2.8±0.13
**Maximal ADP**	[nAO min^-1^mg^-1^]	550±47	509±71	467±65	528±55
**DNP**	[nAO min^-1^mg^-1^]	602±55^§^	573±57^§^	535±94^§^	608±24^§^

The respiratory rate is normalized to mg of mitochondrial protein.

* (P<0.05) W-18wk vs. GK-18 (n = 6)

^§^ (P<0.02) Maximal ADP vs. DNP (n = 6)

### Subsarcolemmal mitochondria

In GK rats (Table 3) at 18 weeks, state 3 respiration rate measured with glutamate is statistically reduced in comparison to the W rats (13.5%). With addition of either a saturating concentration of ADP or by the uncoupler, DNP, the difference is eliminated. In the presence of glutamate and malate, the state 3 rate is not different (wk 18, W: 328±26 vs. GK: 300±25 nAO min^-1^ mg^-1^). In the presence of pyruvate and malate, the respiration rate with a non-saturated concentration of ADP is similar in both groups of rats (Fig 1a), while with a saturating concentration of ADP it is reduced in GK (Fig 2a). Substrates for complex II, III, and IV are used to probe the respective entry points of reducing equivalents into the ETC. In the presence of these substrates, state 3 respiration rate with an unsaturated or saturated concentration of ADP is similar in GK and W rats. Only with a substrate for complex III (Fig 2c) the uncoupled mitochondrial respiration rate in GK is statistically different from that of W rats. State 4 respiration rate measured for different substrate complexes of the ETC is similar in GK and control rats. For the same substrate, RCR is similar in GK and W rats except for a trivial difference for succinate and duroquinol (S1 Fig).

In GK rats at 28 weeks, the respiration rates for glutamate (Table 3) and for pyruvate+malate (Fig 1a) are similar to those of the control rats. In the diabetic group, state 3 respiration rate for complex II, III, and IV substrates with non-saturated or saturated concentrations of ADP are lower (20–25%) than those of the control group (Figs 1a and 2a). Uncoupled oxidative capacity with complex II and III substrates is similar in GK and control rats, whereas with complex IV the rate is lower in GK compared to the control (Fig 2c).

State 4 respiration rate (Fig 1c and 1d) is similar between controls and diabetic rats except that with (TMPD+A)_R_ substrate the respiration rate in diabetic SSM is lower than control group without any change in RCR. Also, state 4 respiration rate increases for complex III substrate from 18 to 28 weeks in diabetic rats with a small change in RCR.

### Interfibrillar mitochondria

In both age groups, the state 3 respiration rate measured with glutamate (Table 3), pyruvate + malate, or substrates for complex II, III, and IV is not different between diabetic and control rats (Fig 1b). Additionally, the respiration rate obtained with a saturated concentration of ADP or by DNP is not altered in diabetic rats at 18 and 28 weeks (Fig 2b and 2d). In diabetic rats, state 4 respiration rates for complex I and III substrates increase from 18 to 28 weeks (Fig 1d), but with no differences between diabetic and control rats.

The ADP/O ratios determined for complexes I, II, and III substrates are similar in diabetic and control rats in both populations of mitochondria at 18 and 28 weeks (S2 Fig). In the progression from 18 to 28 weeks, the ADP/O ratio slightly decreases in both mitochondrial populations in GK with complex I and III substrates.

### Fatty acid oxidation

Mitochondrial fatty acid oxidation was measured in presence of CPT1-dependent (palmitoyl-CoA) and independent (palmitoylcarnitine) substrates. In particular, the enzyme, CPT1, catalyzes palmitoyl-CoA conversion on the outer membrane of mitochondria in the presence of carnitine and the resulting palmitoylcarnitine is transported into mitochondria via carnitine-acylcarnitine translocase. Therefore, oxidative phosphorylation rates measured in the presence of palmitoyl-CoA and palmitoylcarnitine provide information on CPT1 and mitochondrial oxidation of fatty acids. In SSM, fatty acid oxidation in the presence of palmitoylcarnitine or palmitoyl-CoA is similar in diabetic and control rats at 18 and 28 weeks (Fig 3). In IFM, fatty acid oxidation in the presence of palmitoylcarnitine is similar in both rat groups at 18 and 28 weeks while that in the presence of palmitoyl-CoA reveals differences between GK and W rats. At 18 wk, palmitoyl-CoA oxidation is similar in both diabetic and control groups, whereas the oxidation rate increases by 25% from 18 to 28 weeks in IFM from diabetic rats; oxidation does not change in control rats during this period (Fig 3b and 3d). Thus, this adaptation results in a respiration rate 30–35% higher in GK rats than that in the control group at 28 weeks and approaches the oxidation rate of palmitoylcarnitine.

### Electron transport chain and cytochrome assays

**Fig 4:**
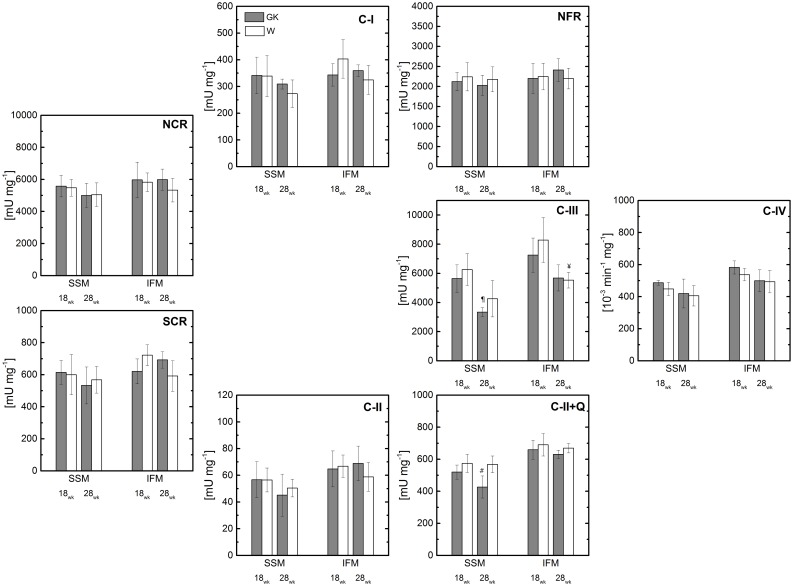
The enzymes activity of ETC of isolated skeletal SSM and IFM at 18 and 28 weeks. Notation as in Fig 1. Rotenone-sensitive NADH-cytochrome c reductase (NCR); NADH ferricyanide reductase (NFR); Antimycin A-sensitive succinate-cytochrome c reductase (SCR); Complex II activity (CII); total complex II with exogenous coenzyme Q (CII+Q); Complex III (CIII); Complex IV (CIV). ^¥^(P<0.05) W-18wk vs. W-28; ^¶^(P<0.05) GK-18wk vs. GK-28; ^#^(P<0.05) W-28wk vs. GK-28; (n = 6), Mean ± SD.

The SSM and IFM function of the ETC complexes is evaluated with specific assays that quantify the activity of the ETC components. The activity of the ETC components in SSM and IFM of diabetic rats are similar to those of the control (Fig 4) group for both age groups. In SSM and IFM, the activity measured for a) rotenone-sensitive CI; b) linked complex I and III (NCR), c) flavin protein domain of complex I (NFR); d) linked complex II and III (SCR) in GK is similar to that observed in controls. In SSM, complex II activity with endogenous coenzyme Q (CoQ) is unaffected, whereas at 28 weeks a minor decrease in activity is observed when exogenous CoQ is added (Fig 4). In IFM, complex II activity with endogenous or exogenous CoQ is unaffected at 18 and 28 weeks. In SSM and IFM, complex III activity is similar in GK and controls at both 18 and 28 weeks; however, in SSM from GK rats there is an aging effect with a decrease at 28 weeks relative to that at 18 weeks. We do not consider as physiologically relevant the differences observed for complex II and III activities because the linked activities of complex I and III (Fig 4, NCR) and of complex II and III (Fig 4, SCR) are unaffected in diabetic rats.

**Fig 5:**
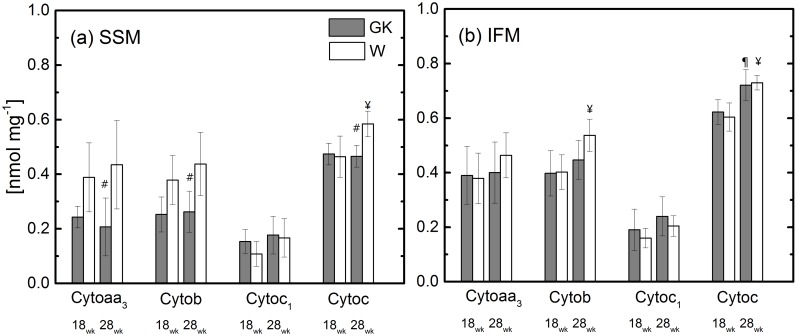
The cytochrome content in isolated skeletal SSM and IFM at 18 and 28 weeks. Notation as in Fig 1. ^¥^(P<0.05) W-18wk vs. W-28; ^¶^(P<0.05) GK-18wk vs. GK-28; ^#^(P<0.05) W-28wk vs. GK-28; (n = 6), Mean ± SD.

In the control group, cytochromes *aa*_*3*_, *b*, and *c*_*1*_ content in SSM are similar to IFM (Fig 5), while cytochrome *c* is higher in IFM than SSM. In the SSM of both control and diabetic rats, the content of most of the cytochromes is similar with age except for cytochrome *c*, where only in the control group, there is a significant increase (26%) from 18 to 28 weeks. At 28 weeks, cytochrome *aa*_*3*_, *b*, and *c* content in SSM of GK rats is significantly lower than that of the control rats. In the IFM population at 18 weeks, no differences are observed between diabetic and control groups for any cytochromes. At 28 weeks, cytochrome *aa*_*3*_, and *c*_*1*_ are not different. Cytochrome *b* and *c* content increases significantly by 32% and 21% in the control group and by 10% and 16% in diabetic rats between 18 and 28 weeks, respectively.

**Fig 6:**
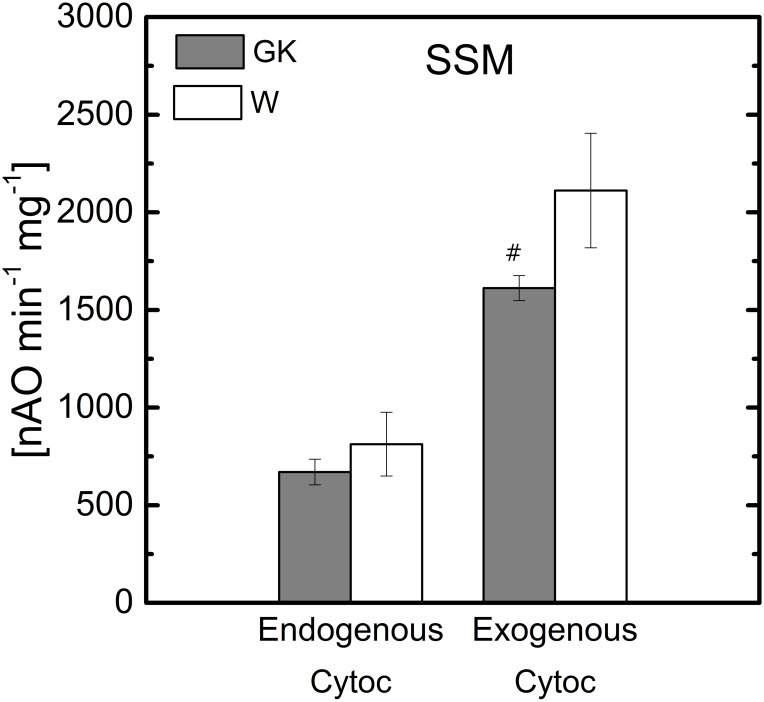
Polarographic cytochrome c oxidase assay of skeletal muscle SSM at 28 weeks. Notation as in Fig 1. ^#^(P<0.05) W-28wk vs. GK-28; (n = 5), Mean ± SD.

The first order rate constant of complex IV measured in solubilized mitochondria (Fig 4) is not different between GK and control rats, but in both coupled or uncoupled SSM the oxidation rate in the presence of complex IV substrate is reduced in GK rats. To reconcile this apparent discrepancy a polarographic assay of azide-sensitive cytochrome c oxidase was performed in permeabilized SSM with endogenous cytochrome c (Fig 6). The assay reveals no differences between GK and W rats. With the addition of exogenous cytochrome c, the activity in both GK and W SSM increases about 2.5 fold; however, a statistically significant decrease of 30% activity is observed in GK rats compared to controls.

## Discussion

An integrated approach that combines oxidative phosphorylation, electron transport chain activity, and biochemical measurements was used to evaluate bioenergetic function in mitochondrial populations of insulin-resistant skeletal muscle from non-obese rats. This approach provided large representative samples of both SSM and IFM populations with a high recovery and high quality from both groups of rats. There were minor defects in oxidative phosphorylation only in SSM while IFM function was preserved. In SSM at 18 weeks, mitochondria dysfunction was confined to a minimally reduced oxidative phosphorylation rate with glutamate, but was normal with a saturated concentration of ADP or in the presence of other substrates of the ETC. In SSM at 28 weeks, the oxidative phosphorylation rate was slightly reduced in the presence of complex II, III, and IV substrates, but is considered unimportant because of the absence of defects upstream as we found with polarographic and spectrophometric assays. Thus, these small deviations do not affect overall SSM bioenergetic function of the diabetic rats. Fatty acid oxidation is unaltered in SSM and IFM at both ages, and palmitoyl-coA oxidation was even enhanced in IFM GK at 28 weeks. These data provide compelling evidence that mitochondrial function is not affected in insulin-resistant skeletal muscle from T2DM non-obese rats.

Our study follows a previous study on the metabolic function of skeletal muscle mitochondria of GK rats evaluated using ^31^P MRS and BOLD MRI [35]. The selfsame GK and control rats used to study mitochondria function *in vivo* [35] were used in this work to study mitochondria *in vitro*. The mitochondrial function in skeletal muscle of GK rats was normal at 12 and 20 weeks and is consistent with our study supporting the absence of impairment in the bioenergetic function of the subpopulations of mitochondria.

The GK rat is a well-characterized non-obese model of T2DM that exhibits spontaneous moderate hyperglycemia and peripheral and hepatic insulin-resistant hyperinsulinemia [36,37] without abnormal elevated content of lipids in blood. In our work, the hyperglycemia and hyperinsulimeia observed in the GK rats at 18 and 28 weeks confirmed the metabolic characteristics of this T2DM model observed in previous work [37, 38, 39].

### Mitochondrial content

A reduced mitochondria content in GK rats was observed only at 18 weeks, while there was no difference between the two groups of rats at 28 weeks. A lower content of SDH in GK compared to controls was previously reported [40]. The animals in the control group continue to grow between 18 to 28 weeks of age while GK rats do not. Consistent with this result, a GK study [41] reported reduced skeletal muscle mass and alteration of muscle fiber distribution, e.g., type I and II, in GK rats. Thus, a skeletal muscle fiber shift from one fiber type to another could be related to the effects observed in the rats at 28 weeks.

### Subsarcolemmal mitochondria

In the presence of glutamate, SSM from GK rat had a lower state 3 respiration rate than that of the control group (Table 3). Oxidative phosphorylation in the presence of glutamate provided information not only on the phosphorylation process, but also on glutamate transport and glutamate dehydrogenase; that in the presence of pyruvate and malate reflects the monocarboxylate and dicarboxylate transporters as well as pyruvate and malate dehydrogenase. The difference between GK and W observed in state 3 respiration in the presence of glutamate should not be ascribed to a defect in complex I because it vanished when a saturated concentration of ADP is used to stimulate oxidative phosphorylation.

The SSM defect observed could be related to the ATP synthase, the adenine nucleotide translocase (ANT) and/or inorganic phosphate transporter. ANT is responsible for ADP/ATP exchange across the mitochondrial inner membrane, while the phosphate transporter provides for the movement of inorganic phosphate into mitochondria. Thus, a reduced respiration rate could be related to a low affinity of ANT for the substrate, but this does not appear to be the case because in our study, the respiration rate with an unsaturated concentration of ADP was not reduced for P+M, G+M, or P-CN. Alternatively, the difference between the two groups could be related to glutamate transporter or glutamate dehydrogenase activity, but this possibility is eliminated because oxidative phosphorylation at saturated concentration of ADP and uncouple respiration rate are unaffected. The difference between GK and W in state 3 respiration rate was eliminated in the presence of glutamate and malate; glutamate is metabolized by aspartate aminotransferase and malate by malate dehydrogenase.

The analysis of the respiration rate in the two populations of mitochondria revealed a specific alteration of the oxidative phosphorylation using substrates for complex II, III, and IV (Figs 2 and 3) only in skeletal muscle SSM of the older GK rats. By the addition of an uncoupler, this defect was relieved when using substrates for complex II and III but was still present using complex IV substrate. The lack of effect on oxidation starting at complex II and III suggests that complex IV does not affect upstream substrates and is physiological irrelevant. The components of the ETC work as units in series with electrons entering in complex I, II and III sites that share the same common path to reach complex IV to reduce oxygen and produce water. Thus, complex IV is located upstream of the ETC and it can control the amount of electrons flowing from complex I to IV per unit of time. In our study, complex I was inhibited with rotenone to evaluate the function of the other complexes. The defect in Complex IV does not limit the electron flux and thus, the respiration rate in presence of complex II or III substrates because the uncoupled mitochondria respiration rate measured in GK rats with complex II or III substrates was similar to that observed in the control group. Furthermore, complex V could be responsible for the respiration difference observed for complex II and III substrates. Under these conditions, the oxidative phosphorylation rates were reduced by 20–25% in comparison to the control group. Although the effect of these mitochondrial alterations on the skeletal muscle remains to be determined, they should not have a major effect on energy metabolism. Indeed, two independent NMR studies reported that mitochondria ATP production was not altered in re-perfused [35] or contracting [42] skeletal muscle at 18 and 28 weeks old GK rats, respectively.

Alternative mechanisms related to a decrease in mitochondrial cytochromes could explain the age-dependent defects in oxidative phosphorylation observed in GK rats at 28 weeks. At this age, significant decreases (50%, 40%, and 16%) were observed in cytochrome *aa*_*3*_, *b*, and *c* in SSM of GK rats compared to the control group. To determine whether cytochrome *c* was responsible for the defect, a polarographic assay of complex IV with and without exogenous cytochrome *c* was performed. The addition of exogenous cytochrome *c* did not correct the defect in the polarographic activity of cytochrome *c* oxidase. This indicates that beside the reduced amount of cytochrome *c*, other factors are responsible for the observed defect.

Cytochrome *b* also was lower than that of the control group (Fig 5). The total cytochrome *b* content is distributed between complexes II and III within the mitochondria [43]. Nevertheless, the reduced cytochrome *b* content in SSM does not affect complex II or III function because the state 3 respiration rate observed for complex II or III substrate was relieved by the addition of the uncoupler (Fig 2a and 2c). Moreover, the activity of complex II and III was unaffected in GK rats (Fig 4). The decrease of the transmembrane protein, cytochrome *aa*_*3*_, which is a subunit of complex IV, is not responsible for the defect observed in SSM of GK rats (Fig 5), because the complex IV assay does not reveal any enzyme activity difference between GK and W rats (Fig 4). It should be noted that the differences between GK and W rats in oxidative phosphorylation rate in the presence of complex IV (Fig 2a and 2c) and endogenous cytochrome c substrates disappear when the mitochondrial membrane is disrupted (Fig 6). This indicates a potential aging effect (from 18 to 28 weeks) leading to a structural defect of the inner mitochondria membrane. A selective aging effect on heart mitochondrial dysfunction was previously reported and attributed to an altered membrane environment, rather than to a reduced protein subunit content [32].

In conclusion, the complex IV defect is not considered relevant because it does not affect the respiration rate obtained with C-II and III substrates and the complex IV enzyme activity assay does not reveal any difference between GK and W rats.

### Interfibrillar mitochondria

The IFM function was normal in GK rats at both age groups. The bioenergetic assays performed to probe the function of the ETC components showed no difference between GK and controls (Figs 1 and 2). In addition, the biochemical assays to quantify the activity of complexes of the ETC showed no difference between GK and controls (Fig 4). This evidence was consistent with the absence of difference in state 3 respiration rate measured in presence of an unsaturated or saturated concentration of ADP and in uncoupled mitochondria (Figs 1 and 2).

### Fatty acid oxidation

Both transport and utilization of fat in myocytes contribute to lipid accumulation. Previous studies presented conflicting results on mitochondrial dysfunction as a key factor in impairment of fatty acid utilization in skeletal muscle cells [4, 7, 8, 44]. Some obese and insulin resistant human and animal studies provided evidence in support of enhanced fatty acid transport [45, 46]. A bioenergetics study on permeabilized skeletal muscle fiber reported on even higher oxidative phosphorylation rate using palmitoylcarnitine in GK rats [38] compared to the control group but similar ADP/O. In that study palmitoylcarnitine respiration rate decreased 5% from 6 to 16 weeks. Thus, the difference between this and our study possibly is related to the GK age.

In our study, the higher respiration rate with palmitoyl-CoA substrate in IFM of GK rats than that obtained for the control group was not related to differences in respiratory capacity of the ETC or mitochondria content since ETC components and CS activities were similar in the two groups. Also, the difference between GK and W rats should not be attributed to an effect of palmitoyl-CoA on ANT, since the palmitoyl-CoA oxidation rate difference between GK and W also was observed with a saturated concentration of ADP (Fig 5). In this condition, the effect of palmitoyl-CoA inhibition on ANT transferase, which is responsible for export/import of ATP/ADP from/to the mitochondrial matrix, is negligible. The difference in palmitoyl-CoA oxidation between the two groups of rats could be related to a higher CPT1 activity in IFM of GK rats than that in IFM of Wistar rats. Although these results are in agreement with enhanced FA transport in obese Zucker rats, the skeletal muscle adaptations appear different between obese and non-obese rats during the development of the disease. While obesity appears to enhance FA transport and oxidation predominately in SSM by an increase of FAT/CD36, CS, and β-hydroxyacyl-CoA dehydrogenase activities with unaltered CPT1 [19], the absence of obesity and presence of T2DM lead to enhancement of FA oxidation only in IFM by CPT-I. It is possible that the enhanced ability to metabolize fatty acid is related to a compensatory skeletal muscle adaptation to the reduced utilization of carbohydrate fuel due to insulin resistance. In T2DM patients and obese insulin-resistant skeletal muscle, CPT-I activity was reduced [47]. The effects of obesity on skeletal muscle metabolic function were also investigated in human subjects [12]. Obesity was found to not alter FA transport and oxidation, while impairment of mitochondrial function was attributed mainly to a reduced content of these organelles.

### Mitochondria efficiency

The ADP/O ratio provides information on oxidative phosphorylation efficiency. The ADP/O ratio is similar in both groups of rats although there is an age effect with a significant decrease of the ratio of both SSM and IFM from 18 to 28 weeks only for GK rats. In human skeletal muscle, the mitochondrial ADP/O ratio of T2DM patients was not different from that of the control group although there was a trend for less efficient mitochondria in the diabetic group [10]. Animal studies showed that skeletal muscle adaptations to obesity are accompanied by an increase of oxidative phosphorylation efficiency [48, 49] that potentially can contribute to the development of insulin resistance induced by a high fat diet [50]. Thus, our study showed a different mitochondrial adaptation to IR in the absence of obesity in comparison to that occurring in obesity.

In conclusion, in the absence of chronic tissue fat overload, the bioenergetic function of both mitochondrial populations of insulin-resistant skeletal muscle is not compromised. Mitochondrial function is normal in T2DM in the absence of a fat overload during the progression of the disease. Thus, increased fuel load rather than mitochondrial oxidative capacity is the trigger event altering insulin action in T2DM [4].

## Supporting information

S1 Fig Respiratory control ratio (RCR) of skeletal muscle SSM (a) and IFM (b) at 18 and 28 weeks. Notation as in Fig 1. Complex I substrate (malate and pyruvate, P); Complex II (succinate and rotenone, S_R_); Complex III (duroquinol and rotenone, DHQ_R_). ^¥^(P<0.05) W-18wk vs. W-28; ^¶^(P<0.05) GK-18wk vs. GK-28; *(P<0.05) W-18wk vs. GK-18 (n = 6); ^#^(P<0.05) control W-28wk vs. diabetic GK-28; (n = 6), Mean ± SD.
(TIF)

S2 Fig ADP to atomic oxygen phosphorylation ratio ADP/O of skeletal muscle SSM (a) and IFM (b) at 18 and 28 weeks. Notation as in Fig 1. Complex I substrate (malate and pyruvate, P); Complex II (succinate and rotenone, S_R_); Complex III (duroquinol and rotenone, DHQ_R_). ^¶^(P<0.05) GK-18wk vs. GK-28; (n = 6), Mean ± SD.
(TIF)

## Abbreviations

ADP: Adenosine diphosphate
ATP: Adenosine triphosphate
BSA: Bovine Serum Albumin
EDTA: Ethylenediaminetetraacetic acid
EGTA: Ethylene glycol tetraacetic acid
KCl: Potassium chloride
KCN: Potassium cyanide
KH_2_PO_4_: Potassium dihydrogen phosphate
KPi: Potassium phosphate
MOPS: 3-(N-morpholino)propanesulfonic acid
