# Supplementary figures and images for: Defects in skeletal muscle subsarcolemmal mitochondria in a non-obese model of type 2 diabetes mellitus
Source: PLoS One. 2017 Aug 29;12(8):e0183978. doi: 10.1371/journal.pone.0183978 (PMC5574550; doi:10.1371/journal.pone.0183978)

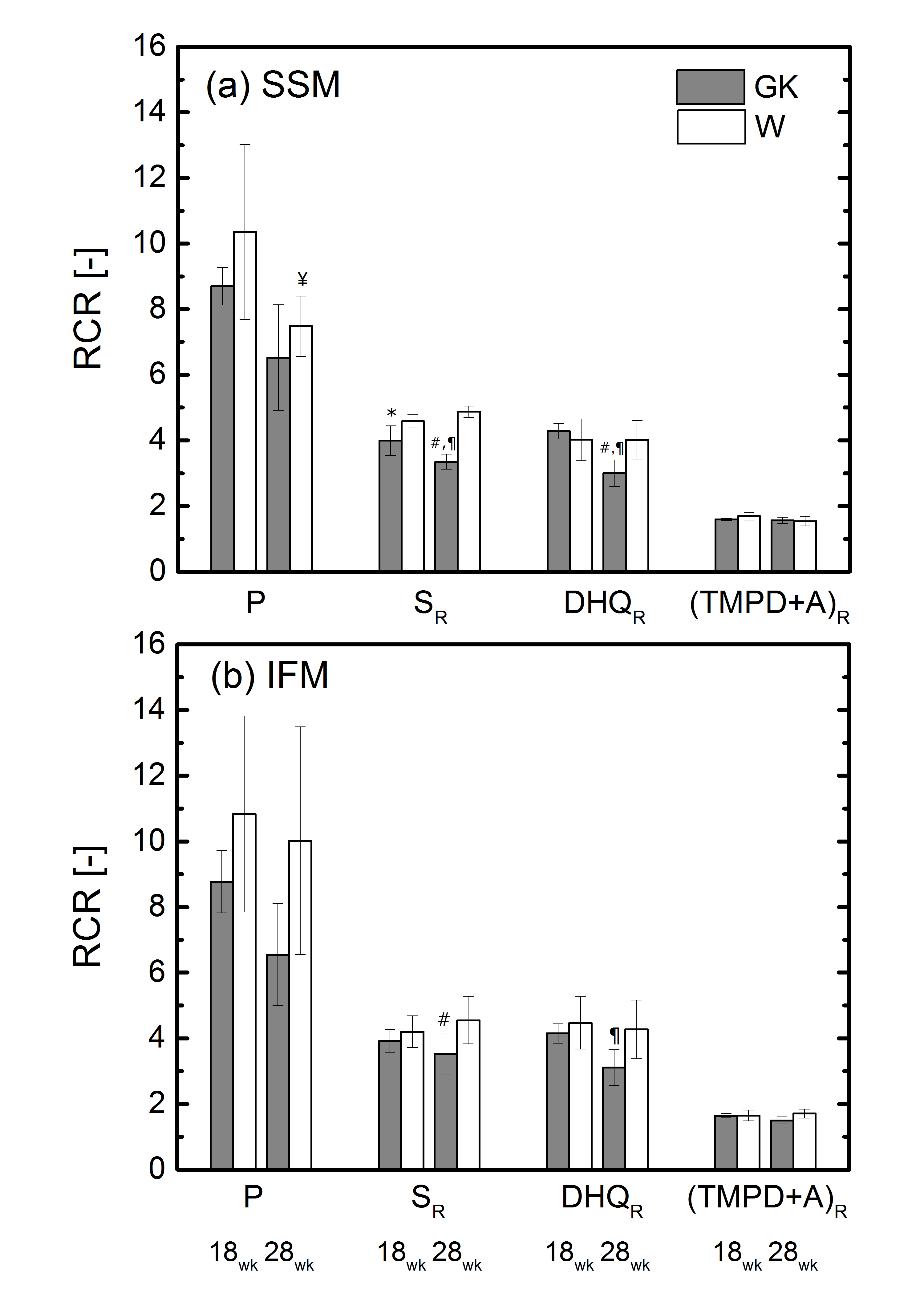

Supplement: S1 Fig — Notation as in Fig 1. Complex I substrate (malate and pyruvate, P); Complex II (succinate and rotenone, SR); Complex III (duroquinol and rotenone, DHQR). ¥(P<0.05) W-18wk vs. W-28; ¶(P<0.05) GK-18wk vs. GK-28; *(P<0.05) W-18wk vs. GK-18 (n = 6); #(P<0.05) control W-28wk vs. diabetic GK-28; (n = 6), Mean ± SD. (TIF) [file pone.0183978.s001.tif]

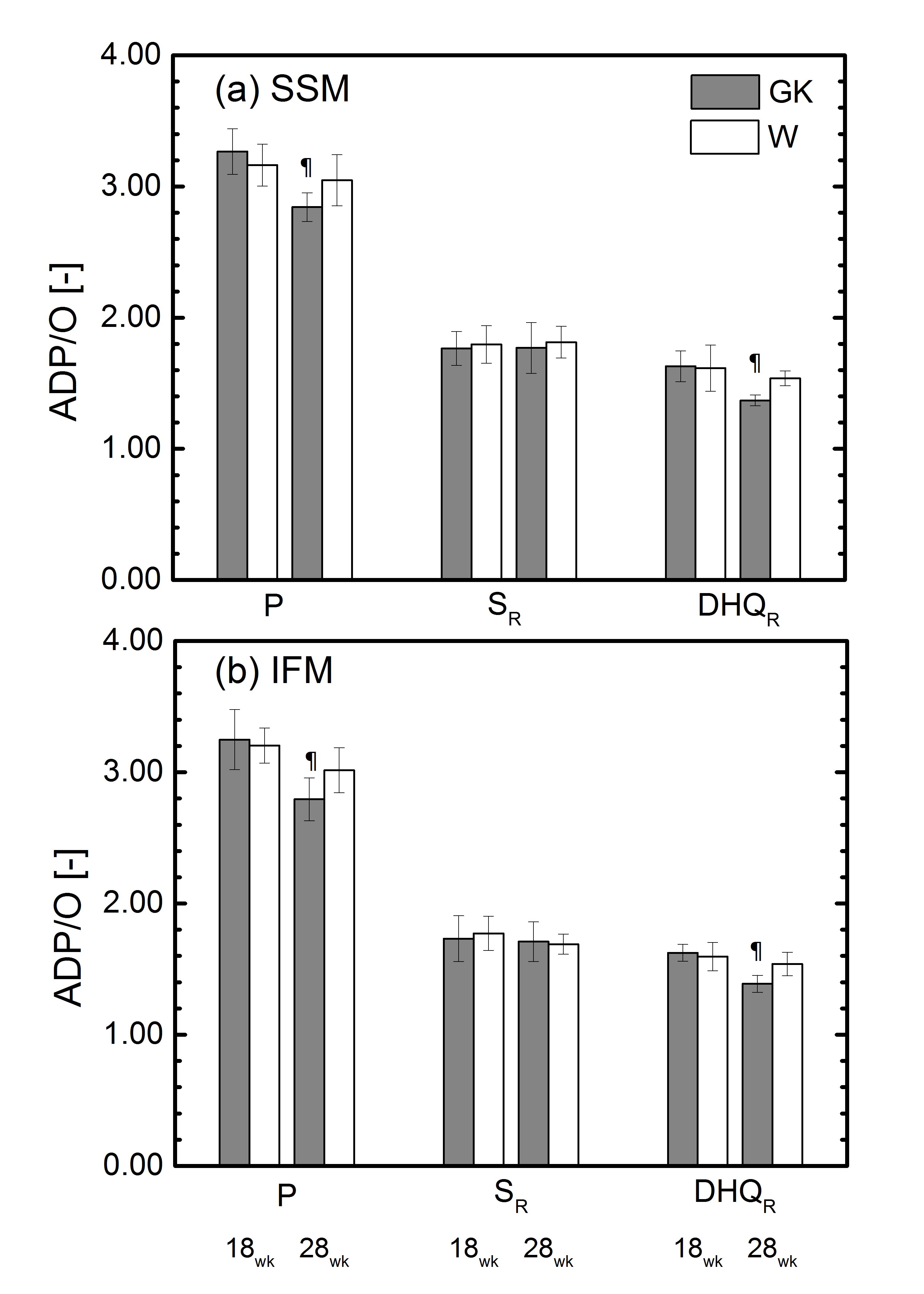

Supplement: S2 Fig — Notation as in Fig 1. Complex I substrate (malate and pyruvate, P); Complex II (succinate and rotenone, SR); Complex III (duroquinol and rotenone, DHQR). ¶(P<0.05) GK-18wk vs. GK-28; (n = 6), Mean ± SD. (TIF) [file pone.0183978.s002.tif]
